# Myths About Intimate Partner Violence Against Women in Becoming a Professional: Influence of Gender and Degree in College Students

**DOI:** 10.3390/bs15060833

**Published:** 2025-06-19

**Authors:** Angeles Rebollo-Catalan, Rafael García-Pérez, Mercedes Cubero-Pérez, Miguel J. Bascón-Díaz, Manuel De la Mata-Benítez

**Affiliations:** 1 Faculty of Education, University of Seville, 41013 Sevilla, Spain; rafaelgarcia@us.es; 2 Faculty of Psychology, University of Seville, 41018 Sevilla, Spain; cubero@us.es (M.C.-P.); mjbascon@us.es (M.J.B.-D.); mluis@us.es (M.D.l.M.-B.)

**Keywords:** intimate partner violence against women, myths, college students, becoming professional

## Abstract

The acceptance of myths about intimate partner violence against women by university students can lead to inappropriate and biased professional interventions due to their gender blindness. The aim of this study is to analyze the acceptance of myths about IPVAW among college students, considering differences by gender and degree course. To do this, we conducted a survey with 1531 first-year college students (55.8% women; mean age 18.89 years) and found that a third of the students did not reject the IPVAW myths and a third of them normalized violence. We also found a higher level of acceptance of violence minimization myths in men than in women, especially in Social Sciences and Engineering. The study reveals the need to educate future professionals about IPVAW myths, with an emphasis on minimization and normalization of violence. It also provides useful information for designing awareness programs.

## 1. Introduction

In this paper, we use the concept of violence against women proposed by the United Nations, which defines it as “any act of gender-based violence that results in, or is likely to result in, physical, sexual or psychological harm or suffering to women, including threats of such acts, coercion or arbitrary deprivation of liberty, whether occurring in public or in private life” (United Nations, 1993). One of the expressions of this type of violence is intimate partner violence against women (IPVAW), which alludes to all acts of physical, sexual, psychological, or economic violence perpetrated by a current or former intimate partner, regardless of whether or not the perpetrator shares or has ever shared a joint residence with the victim (EIGE, 2023; Paz-Rodríguez et al., 2022). As some previous studies have shown (Ferrer-Pérez et al., 2019; Herrero et al., 2017), IPVAW constitutes a type of gender-based violence, since its aim is to exercise power and domination over women through violent coercive control, which in turn is an expression of the structural inequality inherent to patriarchal societies, in whose origin sexist beliefs and misogyny play a key role. In this sense, our study focuses on the acceptance of myths about intimate partner violence against women, which, as other studies have shown (Albertín-Carbó et al., 2020; Badenes-Sastre et al., 2023; Noriega et al., 2020; Davies & Berger, 2019), can lead to biased and inappropriate professional interventions.

Intimate partner violence against women is a public health issue that adversely affects the health of women and is one of the most pervasive human rights violations in the world, rooted in gender inequality, discrimination, and harmful cultural and social norms (World Health Organization [WHO], 2021). Some reports also highlight the increasingly young age at which women experience their first abusive relationship (DGVG, 2020; Díaz-Aguado et al., 2021; Ruiz-Repullo, 2016). Moreover, research has also shown that adolescents and young women tend to normalize the initial signs of abuse (DeVault, 2019; Nardi-Rodríguez et al., 2017; Rebollo-Catalan & Mayor-Buzon, 2020), precisely due to their beliefs about couple relationships and gender-based violence.

We now know that social discourses which justify violence, blame the victim or exonerate the perpetrator have a negative influence on victims, increasing their vulnerability and lack of support (Lim et al., 2015; Yamawaki et al., 2012). We also know that the higher the myth acceptance, the lower the likelihood of intervening in cases of intimate partner violence against women (Murvartian et al., 2025).The growing rise of political forces that deny the very existence of violence against women and question institutional policies to support the victims, with a great impact on public opinion (Delgado-Ontivero & Sánchez-Sicilia, 2023; Ramis-Moyano et al., 2023), makes the investigation of false beliefs about IPVAW in college students even more necessary, as these false beliefs may condition their future professional activity.

For all these reasons, we carried out the present study with the objective of detecting the acceptance level of IPVAW myths among college students.

### 1.1. Myths About IPVAW

The perspective adopted in this study, as well as our view of the implications of these myths for the maintenance of IPVAW, are rooted in the definition offered by Peters (2008), for whom myths are “stereotypical beliefs about domestic violence that are generally false but are widely and persistently held, and which serve to minimize, deny, or justify physical aggression against intimate partners” (p. 6). These types of myth are defined as false beliefs that are at the basis of the justification of violent behaviors and attitudes toward women.

Bosch-Fiol and Ferrer-Pérez (2012) reviewed and updated the myths identified by Peters (2008), finding four types: myths about the marginal or exceptional nature of IPVAW (e.g., gender-based violence only occurs in immigrant or low-income families and individuals); myths about perpetrators that in some way exonerate them from blame (e.g., perpetrators are mentally ill, or they consume/abuse alcohol and/or drugs); myths about abused women that hold victims accountable for the violence perpetrated against them (e.g., some women with certain characteristics are more likely to be victims, or women who experience gender-based violence do something to provoke it); and myths that minimize the seriousness or importance of violence against women. Among them, the myths that minimize the severity of IPVAW or even deny it (most reports of victim women are false; men are also victimized; laws criminalize men) are particularly significant because they question the need for and the usefulness of positive action plans, programs, and measures targeted at victims, increasing their vulnerability and lack of institutional protection.

In a study carried out on the Spanish population aged 17 to 81, Ferrer-Pérez et al. (2016) found that around a third of the people surveyed had false beliefs about those who suffer or perpetrate such violence or about the existence of false complaints, and, in general, this proportion prevails, regardless of sex, age, or study level, concluding the need to continue working on training and social awareness about this violence. Furthermore, Megías et al. (2018) found that men scored significantly higher than women on acceptance of IPVAW myths in adult populations from Spain and the United States. These findings were also found by Gracia et al. (2020) and Sánchez-Prada et al. (2022).

Other studies (Ballesteros et al., 2018; Del Moral et al., 2020) conducted with young people have found that 27.4% believe that it is normal in couple relationships and 21.2% think that it is an exaggerated politicized issue and have observed a higher degree of acceptance among boys than among girls, so they conclude the need to develop educational programs specially designed for men.

According to Rodríguez-Martín et al. (2020), the justification of IPVAW has increased more than 20 points among young people in the last decade. In this regard, the authors found that 46.6% of the participants in 2019 responded that a large number of men who attack their partner are ill or drink too much, compared to the 22.9% that held a similar opinion in 2010. Additionally, 63.9% of the participants in the last study claimed that both women and men are equally victims of intimate partner violence (compared to 43.7% with a similar opinion in 2010). In this sense, Del Moral et al. (2020) also found that 90.8% of the participants (males and females) did not agree with the statement “Intimate Partner Violence only occurs when a man assaults a woman and not the other way around, that is, they maintain a definition in which this kind of violence can occur from a man to a woman, but also from a woman to a man” (p. 9). This belief is particularly significant because, as Fernández-Zurbarán (2018) stated, bidirectional violence makes it difficult for professionals to detect IPVAW, especially when it occurs among adolescents and young people. This belief equates violence with aggression and erases the instrumental nature of violence as a tool to exert control and dominance over women.

The normalization of IPVAW has been understood as something more than a means of minimizing or justifying this type of violence (Lim et al., 2015; McCarry & Lombard, 2016; Waltermaurer, 2012). Rodelli et al. (2022) consider the normalization of gender-based violence against women as those cultural beliefs and values that support and justify the perpetration of gender-based violence by presenting it as a normal component of relationships between men and women. Some of these beliefs hold that an abuser can be a good friend (Sinko & Saint Arnault, 2020) or a good father (Procentese et al., 2020). Some studies have found that many young people fail to identify early warning signs of violence against women, labeling them as “normal” (Del Moral et al., 2020; DeVault, 2019; Nardi-Rodríguez et al., 2017). Thus, the inability to recognize signs of coercive control behavior ends up legitimizing the maintenance of power over women and increases victims’ vulnerability and lack of protection (Lim et al., 2015; Yamawaki et al., 2012).

### 1.2. Myths About IPVAW in Professionals and Future Professionals

The existence of myths about IPVAW in public service professionals is particularly worrying for its negative consequences on their interventions with women victims of IPVAW. For this reason, it is mandatory to train professionals in their responsibilities. Numerous studies have shown that gender prejudices and stereotypes hinder effective intervention in situations that require sensitivity and specialized knowledge—such as detecting cases of gender-based violence, assessing and managing risk, and preventing secondary victimization. These limitations are evident across various sectors, including healthcare (Badenes-Sastre et al., 2023; Noriega et al., 2020), the justice system (Albertín-Carbó et al., 2020), and education (Davies & Berger, 2019). In this area, some studies have shown that training and awareness of gender issues improve professional competence (García-Pérez et al., 2013; Murvartian et al., 2025).

Sensitivity and training deficits regarding gender equality for professionals who may intervene in IPVAW cases make it necessary not only to train practicing professionals, but especially, future professionals, as well. With regard to this question, the Europe Horizon (2021) program promotes actions aimed at developing professional skills in this area, emphasizing the importance of education. On the other hand, Spanish legislation calls for the social responsibility of universities in the training and qualification of professionals sensitive to gender equality, especially in the Social and Legal Sciences and Health Sciences, making an explicit mention of professionals in the field of education. For this purpose, it has established the obligation to include transversal content or competences related to gender equality in university degrees (Real Decreto 822/2021, 2021). However, as Ballarín (2017) notes, changes within universities have been very slow, reflecting the persistence of patriarchal thinking and practices within the institution. Universities continue to operate through mechanisms and symbols that reinforce male dominance in both knowledge production and teaching. This is the case despite efforts by Spanish universities—through their Gender Equality Units—to develop equality plans that prioritize awareness-raising and gender equality training as a central focus (Pastor-Gosálbez et al., 2020). It is therefore necessary to eliminate the deficit of knowledge about women and about gender relations in higher education to build and teach forms of knowledge that do not discriminate or maintain the subordinate character of women or any other marginalized group (Pérez-Sedeño, 2018).

### 1.3. Acceptance of Myths About IPVAW and Sexist Attitudes

Research has also demonstrated the relationship between sexist attitudes and violence against women (Hammond & Overall, 2015; Valor-Segura et al., 2011). Thus, some studies have reported a close association between justification of violent behavior and high scores for sexist attitudes, with a greater likelihood of perpetrating violence in the university context (Erdem & Sahin, 2017; Rivas-Rivero & Bonilla-Algovia, 2024). Although this population has been found to have lower levels of sexism than other groups (Ferrer-Pérez et al., 2019; Murvartian et al., 2025), rendering these attitudes visible and eradicating them is particularly important due to the possible negative impact they may have on their future professional practice (Albertín-Carbó et al., 2020; Badenes-Sastre et al., 2023; Davies & Berger, 2019; Noriega et al., 2020).

Additionally, some studies have found differences in the acceptance of myths about violence against women and sexist attitudes related to the course of the degree. For instance, Esteban-Ramiro and Fernández-Montaño (2017) found that more sexist opinions were expressed in Business Administration and Management (University of Castilla La Mancha, Spain) than in Social Work and Social Education. Moreover, in Law and Humanities in northern Mexico, a higher degree of acceptance of beliefs about sexual assault was found than in Medicine or Psychology (Bernal-Baldenebro et al., 2019).

Based on results from the previous research mentioned above (Bosch-Fiol & Ferrer-Pérez, 2012; Ferrer-Pérez et al., 2019; Peters, 2008), the aim of this study was to determine to what extent myths about IPVAW are accepted among university undergraduates, analyzing differences related to gender and degree course. Ultimately, we seek to determine what types of myths are most accepted by students, which could be key content in IPVAW awareness programs in the university context.

Given that previous studies have found differences in the sexist attitudes held by students in accordance with degree course, while at the same time have confirmed the link between ambivalent sexism (hostile and benevolent) and IPVAW, we aimed to compare the level of acceptance of myths about IPVAW across different university disciplines. This may help identify groups that are at greater risk, allowing targeted interventions.

## 2. Method

### 2.1. Participants

The sample consisted of 1531 first-year students at a public university in the south of Spain. All participants were between 18 and 24 years of age (*M* = 18.78; *SD* = 1.03) and 55.8% were female. Four participants did not declare their gender and seven did not identify themselves as men or women.

The sample was selected using a proportional stratified random sampling by clusters. The size of the sample allowed us to work with a sampling error of ±2.5% for a confidence level of 95.5%. The students were recruited from various degree courses that corresponded to different fields of study: 18.5% studied Health Sciences, 47.4% Law and Social Sciences, 17% Natural Sciences and Mathematics, and 17.1% Engineering. All students in the sample were in the second semester of the first year of their degree courses. It was a voluntary decision to recruit first-year students in order to provide valuable information for the gender equality assessment conducted annually at Spanish universities. Understanding the false beliefs about IPVAW among incoming students can help incorporate action measures into universities’ equality plans and also contribute to raising awareness among faculty and academics about their responsibility to provide quality training to incoming students in this subject, leading to improved professional performance.

### 2.2. Instruments

*Myths about the Intimate Partner Violence against Women Scale (MIPVAW)* (García-Pérez et al., 2024). This comprises 15 items rated on a Likert-type scale (1 = completely false; 2 = false; 3 = neither true nor false; 4 = true; 5 = completely true) and measures four dimensions: (a) violence minimizing, which includes four items (7, 9, 13, and 14) that express ideas that reduce the seriousness of IPVAW and even deny its existence, questioning institutional policies in support of victims (α = 0.74); (b) blaming the victim, which includes four items (2, 4, 5, and 8) that show ideas that hold victim women responsible for what happens to them, shifting the blame from the abuser to the victim (α = 0.66); (c) exonerating the perpetrator, which includes four items (1, 3, 6, and 15) that emphasize personal factors that lead a man to abuse his partner and exonerate him of guilt (α = 0.62); and normalizing violence, which includes three items (10, 11, and 12) that express the idea that a man who abuses his partner can be a good friend, co-worker, or good father (α = 0.66). These values above 0.60 and up to 0.70 are acceptable according to the standards used for research (Lloret-Segura et al., 2014). The alpha coefficient for the global scale was 0.80. This four-factor structure of the MIPVAW scale has been verified by Exploratory and Confirmatory Factor Analysis. (a) EFA (CATPCA: total eigenvalue = 7.97; Cronbach’s α = 0.94). All elements had factor loadings higher than 0.50 in their corresponding factors. (b) CFA (χ^2^ = 149: df = 79; χ2/df = 1.89; *p* < 0.001) with good fit indicators CFI = 0.98, TLI = 0.98, SRMR = 0.024, and RMSEA = 0.024, with the 90% CI [0.018, 0.030]. Concurrent Validity was calculated with ambivalent sexism (*rho* = 0.60; *p* < 0.001), benevolent sexism (*rho* = 0.46; *p* < 0.001), and hostile sexism (*rho* = 0.68; *p* < 0.001).

*Sexist Attitudes*. We used the 22-item Ambivalent Sexism Inventory (Glick & Fiske, 1996; Spanish version by De Lemus et al., 2008), which assesses two dimensions, with 11 items for each scale: hostile sexism and benevolent sexism. All items are rated on a 5-point Likert-type scale, ranging from 1 (completely disagree) to 5 (completely agree), with higher scores reflecting higher levels of sexism. In the current study, internal consistency reliability was good: 0.93 for hostile sexism, 0.87 for benevolent sexism, and 0.94 for ambivalent sexism.

### 2.3. Procedure

Once we determined the sample size, we contacted university professors who were teaching the different degree courses during the second semester of the first year to arrange the day to administer the questionnaire during class time. The questionnaire was collectively administered to all the students attending the class on that day.

The students were informed about the objectives of the study and assured that participation was voluntary and anonymous. No compensation of any kind was offered in exchange for participation. Those who agreed to participate completed an informed consent form followed by a questionnaire in pen and paper format. In addition to guaranteeing confidentiality of the data provided, we also informed the participants that they could withdraw from the study at any time, simply stating their wish to do so. The mean time taken to administer the questionnaire was 20 min in each group. The study was carried out in accordance with current ethical standards, and its design was approved by the University Ethics Committee.

### 2.4. Data Analysis

Once the data was entered into the SPSS statistical package version 24 for Windows, an exploratory analysis of the variables was performed to determine their structure and distribution. Once the validity of the construct of the IPVAW myth acceptance scale was verified, we created a variable from the sum of all the items, leaving a global variable for acceptance of myths about IPVAW on a scale from 15 to 75, and four variables on a scale from 4 to 20 for the dimensions of violence minimizing, blaming the victim, and exonerating the perpetrator, and from 3 to 15 for the dimension of violence normalizing.

**Figure 1:**
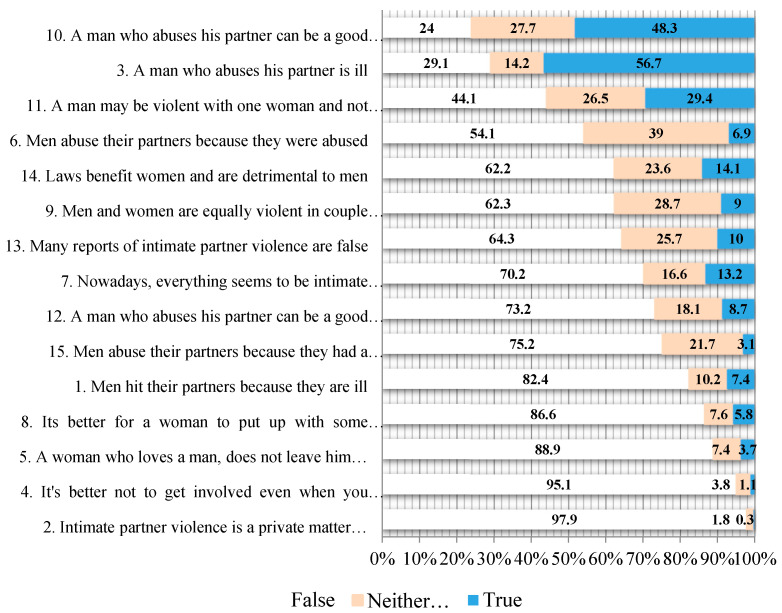
Acceptance level of myths about IPVAW for the whole sample. The full text of the items can be found at: García-Pérez et al. (2024).

We present the distribution of IPVAW myth acceptance percentages on a three-point scale (false, neither true nor false, and true) in Figure 1, having first tested that the recoding did not affect either the validity or the reliability of the scale. This recoding consisted of grouping values 1 and 2 (false and completely false) and 4 and 5 (true and completely true) of the scale together and was done in order to enable the complete yet summarized presentation of the responses obtained in all items.

Pearson’s r was used to analyze the relationship between the level of acceptance of myths about IPVAW and sexist attitudes (hostile and benevolent sexism). We used the sexism measures only to study the concurrent validity of the IPVAW myth acceptance scale. Consequently, we present only the correlations between the sexism measures and IPVAW myth acceptance.

To test the difference hypothesis, we applied Student’s *t*-test and ANOVAs. Also, we ran a two-factor ANOVA using a General Linear Model to study the interaction between gender and degree course, with partial *η*^2^ values giving 0.01 (small), 0.09 (medium), and 0.25 (large). To calculate the effect size, we applied Cohen’s d, with values around 0.20 indicating a small, around 0.50 a medium, and 0.80 a large effect. The post hoc tests were analyzed by the Tukey honest significant difference (HSD) method (*α* = 0.05).

Eleven students were excluded from the correlation and contrast analyses that required the gender variable because they did not declare their gender or did not identify as men or women. We believe that those who did not identify as men or women would be the subject of a specific study on non-binary groups, which goes beyond the focus and objective of this research.

## 3. Results

### 3.1. Acceptance of Myths About IPVAW

The results revealed significant correlations greater than 0.30 between all myths and sexist attitudes (Table 1), except between the normalizing myths of violence and benevolent sexism, where the correlation was very low between women, *r* (n = 848) = 0.15, *p* < 0.001, and men, *r* (n = 672) = 0.01, *p* < 0.05. Nevertheless, the correlation between minimizing violence and hostile sexism was particularly strong, both among women, *r* (n = 848) = 0.60, *p* < 0.001, and among men, *r* (n = 672) = 0.75, *p* < 0.001.

Participants scored below the mean of the scale for all variables, with the lowest scores recorded for blaming the victim, between women (*M* = 1.23, *SD* = 0.39) and men (*M* = 1.41, *SD* = 0.49); and the highest scores recorded for normalizing violence, again between women (*M* = 2.52, *SD* = 0.86) and men (*M* = 2.76, *SD* = 0.90).

An analysis of the responses to each item on the scale provides a more detailed overview (Figure 1).

Three items reflect the highest level of acceptance by students. Thus, more than half of the students claimed to accept the idea that perpetrators of IPVAW are ill (Item 3). Furthermore, almost half of the respondents believe that a man who abuses his partner can be a good neighbor, friend, or work colleague (item 10), and almost a third of them consider it is true that a man can be violent with one woman and not with another (Item 11), a belief that, in addition to normalizing violence as a legitimate means for men to relate to women within a couple relationship, also shifts the responsibility for the abuse from the perpetrator to the victim.

It is important to note the high percentage of students who adopted an intermediate position in response to some items on the scale. This group of items includes false beliefs about the legislation that protects victims (many reports of gender-based violence are false; the law benefits women; etc.). Also striking is the fact that almost a third of the students adopted a neutral attitude to the statement that men and women are equally violent in couple relationships (item 9), and a high percentage said that they considered men abuse their partners because they themselves were abused as children (item 6) to be neither true nor false.

Figure 1 also reveals the almost unanimous rejection by university students of myths that gender-based violence is a private affair between the members of the couple and that it is best not to get involved (items 2 and 4).

### 3.2. Acceptance of Myths About IPVAW by Gender

We found statistically significant differences in overall acceptance of myths according to gender (Table 2), with a moderate effect size, *t* (14) = 12.00, *p* < 0.001, *d* = 0.65. However, these differences were mainly focused on myths about minimization of violence, *t* (1126) = 15.17, *p* < 0.001, *d* = 0.37, with more men than women believing these myths to be true. In this sense, we should highlight item 7, which claims that IPVAW is overly exaggerated today; 23.3% of men believed this statement to be true, compared to 5% of women, with a large effect size, *t* (1288) = 15.52, *p* < 0.001, *d* = 0.82. Also worth noting is item 14, which states that laws benefit women and are detrimental to men. In our sample, 21.7% of men believed this to be true, compared to 8.1% of women, with a moderate effect size, *t* (1343) = 11.84, *p* < 0.001, *d* = 0.62.

The results also revealed gender differences in the acceptance of myths linked to blaming the victim, although the effect size was small, *t* (1227) = 7.41, *p* < 0.001, *d* = 0.19. These differences were mainly concentrated in item 5, which claims that a woman who loves her partner does not leave him, even if he is violent, *t* (1229) = 7.20, *p* < 0.001, *d* = 0.39. However, these differences were small and focused on intermediate positions on the response scale, with a higher percentage of women adopting a firm position of disbelief (completely false 86.5%, false 6.0%, neither true nor false 4.4%, true 2.6%, and completely true 0.5%) than men (completely false 66.3%, false 18.1%, neither true nor false 11.1%, true 3.6%, and completely true 0.9%).

### 3.3. Acceptance of Myths About IPVAW by University Degree

Statistically significant differences were found in the general acceptance of myths depending on the degree course, with moderate effect size, *F* (3) = 14.94, *p* < 0.001, *d* = 0.51. However, the results also revealed that these differences were mainly concentrated in myths related to the minimizing of violence, *F* (3) = 16.81, *p* < 0.001, *d* = 0.56, specifically items 7 and 14, for which the effect size was moderate. Significant differences were also found in myths related to victim blame, with a moderate effect size, *F* (3) = 8.24, *p* < 0.001, *d* = 0.40. These differences were mainly concentrated on items 4 and 5.

In the post hoc pairwise comparison, we applied Tukey’s honest significant difference (HSD) method to every dimension score (minimizing violence, blaming the victim, exonerating the perpetrator, and normalizing violence) and the overall MIPVAW score. All tests agreed that myth acceptance formed two subsets (α = 0.05), with the Engineering group forming a subset with a significantly higher average compared to the subset made up of all the other degrees.

The results of a two-factor ANOVA revealed a small effect of gender on myth acceptance depending on the degree course, indicating that the differences observed between degree courses in relation to minimizing violence were slightly influenced by gender, *F* (1, 3) = 4.42, *p* = 0.004, *η*_p_^2^ = 0.009. However, this effect was not observed for myths linked to blaming the victim, *F* (1, 3) = 1.87, *p* = 0.133, *η*_p_^2^ = 0.004, exonerating the perpetrator, *F* (1, 3) = 0.22, *p* = 0.88, *η*_p_^2^ = 0.000, or normalizing violence, *F* (1, 3) = 0.25, *p* = 0.859, *η*_p_^2^ = 0.001.

The gender differences in the acceptance of the IPVAW myth within each field of study showed that in all degree programs, the differences between men and women occurred in relation to myths about the minimization of violence, with a moderate effect size in Health Sciences, *t* (274) = 4.39, *p* < 0.001, *d* = 0.60, and Natural Sciences and Mathematics, *t* (239) = 3.947, *p* < 0.001, *d* = 0.50; and a large effect size in Engineering, *t* (75) = 6.297, *p* < 0.001, *d* = 0.95, and Law-Social Sciences, *t* (396) = 10.95, *p* < 0.001, *d* = 0.89. The results also revealed gender differences in the acceptance of myths related to blame among Engineering students with a moderate effect size, *t* (254) = 2.733, *p* = 0.007, *d* = 0.47, and students of Law-Social Sciences, *t* (379) = 4.95, *p* < 0.001, *d* = 0.40, and Health Sciences, *t* (96) = 2.42, *p* = 0.017, *d* = 0.35, both with a small effect size. We found no gender differences in acceptance of myths about blaming victim among students in Natural Sciences and Mathematics.

When we analyzed the data separately for men and women to explore differences in myth acceptance associated with the degree course, we found no differences among women depending on the degree course in relation to minimizing violence, *F* (3) = 1.56, *p* = 0.196, *d* = 0.18, and blaming the victim, *F* (3) = 0.29, *p* = 0.834, *d* = 0.10, although differences were observed in these dimensions among men, *F* (3) = 4.98, *p* = 0.002, *d* = 0.41, in violence minimizing, and *F* (3) = 3.62, *p* = 0.013, *d* = 0.42, in blaming the victim, with, respectively, those studying Engineering and Law-Social Sciences showing the highest levels of acceptance. This indicates that the differences observed depending on degree course are mainly due to higher levels of acceptance among men of the myths related to violence minimizing and victim blaming on these courses.

## 4. Discussion

The aim of the study was to determine the level of acceptance of myths about IPVAW among university undergraduates, due to its relevance for future professional practice. In this sense, we have analyzed the differences according to gender and degree course. In general, the results reveal a low level of acceptance of IPVAW myths among the participants in our study. This finding is consistent with those reported by Ferrer-Pérez et al. (2019), who observed that the level of acceptance of these beliefs among university undergraduates is lower now than a decade ago. These researchers found that women’s acceptance of myths had decreased overall, but they also found less acceptance of beliefs about women’s inferiority among men, which they attributed to the fact that awareness-raising measures adopted in Spain (e.g., legislative changes, prevention campaigns, or academic training programs) had an effect on previously held beliefs.

However, the overall improvement observed in relation to raising public awareness of IPVAW and making it more visible is not evenly distributed. In this sense, as has been reported in fact by other authors (Ballesteros et al., 2018; DeVault, 2019; Rodríguez et al., 2023), our data reveal a higher level of acceptance of myths related to normalization of violence and exoneration of the perpetrator.

Thus, we observed a high level of acceptance of certain myths. This is the case with the idea that perpetrators of IPVAW are ill (item 3), that they can be good neighbors, friends, or work colleagues (item 10) and that men may be violent with one woman but not with another (item 11). These ideas were widely accepted among the students in our study, both male and female, a finding that suggests that those men who are violent with their partners can retain their reputation and social support. As some authors have pointed out (Borrajo et al., 2015; Erdem & Sahin, 2017; Sánchez-Prada et al., 2022), a greater acceptance of these myths increases perpetrators’ tendency to engage in and legitimize violent behavior, hence showing the importance of training young men to recognize the erroneous nature of these beliefs. Furthermore, accepting the belief that the perpetrators are considered ill means that they are deemed worthy of care and attention. As previous research has stressed (Nardi-Rodríguez et al., 2017; Paz-Rodríguez et al., 2022; Peters, 2008), this belief is particularly harmful to women, as it leaves them unprotected and makes them more vulnerable to abuse. Our results suggested that the myths linked to normalizing violence are precisely those most accepted among first-year university students, suggesting that they should be a priority focus for prevention plans.

The fact that a high percentage of women claimed to accept these beliefs means that it is particularly important to provide them with training on these issues, to enable them to detect early warning signs and offer tools to intervene. As we have pointed out before, previous studies have stressed the fact that young men and women tend to consider these early signs of violence as normal (Rebollo-Catalan & Mayor-Buzon, 2020; Ballesteros et al., 2018; Del Moral et al., 2020; Nardi-Rodríguez et al., 2017). Our results revealed that training actions targeting college women are necessary to help them identify and reject IPVAW myths, with special emphasis on those linked to normalizing violence and exonerating the perpetrator, not only as a strategy for self-protection, but also as a means of enabling them to act in a timely and efficient manner later on in their professional lives.

However, we would also like to highlight the high percentage of students who adopted an intermediate position, neither accepting nor rejecting the myths of violence minimization, particularly those associated with the legislation designed to protect victims (many reports are false; laws benefit women; etc.). This intermediate position was also observed in relation to the belief that men and women are equally violent in couple relationships. As some experts have warned (Fernández-Zurbarán, 2018; Paz-Rodríguez et al., 2022; Ruiz-Repullo, 2016), cross-violence among adolescents and young people all too often conceals a story of intimate partner violence against women, making it harder to diagnose and take early action. Some studies (Del Moral et al., 2020; Rodríguez-Martín et al., 2020) have shown an increase in the acceptance of the belief that men and women are equally violent in couple relationships among young people. This kind of ambiguous response by students seems to indicate a certain lack of knowledge, suggesting that future interventions aimed at preventing IPVAW should take this into account.

In relation to gender, our results revealed significant differences in the level of acceptance of IPVAW myths, with men tending to accept them more than women. These results are similar to those found in other studies (Del Moral et al., 2020; Ferrer-Pérez et al., 2019; Gracia et al., 2020; Herrero et al., 2017; Megías et al., 2018; Sánchez-Prada et al., 2022), in which men were found to have higher levels of acceptance of IPVAW myths than women. In our study, this is particularly evident in relation to myths linked to minimizing violence. This is important because, as some previous studies have shown (Erdem & Sahin, 2017; Waltermaurer, 2012), it is not just that men who hold these beliefs are often incapable of identifying the signs of intimate partner violence against women, it is also that they themselves are more likely to legitimize and even perpetrate it. Over a decade ago, Ferrer-Pérez et al. (2006) highlighted the importance of including IPVAW awareness training in the university curriculum, particularly in those disciplines responsible for training the future professionals who will one day work in this area. They said this because it was male university students with no training in IPVAW who, over ten years ago, were found to have a greater tolerance for intimate partner violence against women, a greater acceptance of violence as an adequate means of resolving conflicts, and a greater tendency to minimize the seriousness of this kind of violence. Our results may be encouraging and point to the positive effect of the awareness-raising and prevention measures implemented to reduce the level of acceptance of victim-blaming myths among university men. However, social discourses from the manosphere and far-right forces that minimize violence against women and question affirmative action policies for victim care appear to influence the acceptance of violence-minimizing myths among university men (Delgado-Ontivero & Sánchez-Sicilia, 2023; Ramis-Moyano et al., 2023). This is particularly worrying among those who will practice their profession in the legal, educational, or healthcare systems due to their responsibility in providing victim care services (Albertín-Carbó et al., 2020; Badenes-Sastre et al., 2023; Davies & Berger, 2019; Noriega et al., 2020). These results suggest the need to include violence minimization as part of awareness-raising campaigns and programs in the university community, with actions particularly directed at university men.

Regarding the field of study, our results revealed differences in the level of acceptance of IPVAW myths between degree courses, particularly in terms of acceptance of myths that minimize violence, with those studying Engineering and Law-Social Sciences degrees having higher scores in this area. This difference can be partially explained by the proportion of male and female students across courses. However, we still found higher acceptance of these myths among male students from Engineering and Law-Social Sciences courses than among their counterparts studying other degrees. These findings suggest the need to channel the training provided to men towards myths linked to minimizing violence, focusing particularly on the Engineering and Law-Social Sciences courses, which are especially vulnerable to beliefs that seek to accept and legitimize intimate partner violence against women. These results are similar to those found in other studies (Bernal-Baldenebro et al., 2019; Albertín-Carbó et al., 2020), that found greater acceptance of myths in the law and humanities professionals than in psychology and medicine. However, our study identifies engineering students as the target group for prevention and awareness-raising, representing a group of particular interest due to their involvement in the development of digital security products and services. Epifanio and Calvo-Iglesias (2024) already point this out as a shortcoming of equality plans of most Spanish universities, which lack strategic lines for including gender mainstreaming in STEM disciplines.

### Limitations and Further Research Directions

This study has some limitations. Firstly, our sample consisted of university students from a single region in southern Spain, which limits the generalizability of the results and leads us to take these findings with caution until further research could be conducted with more heterogeneous samples. Secondly, the results were based on self-report questionnaires only, so it would be advisable to carry out qualitative studies that provide us with more information about how thinking is distorted by these myths and what is behind the answers to these questionnaires. Finally, we did not measure the potential distorting effect of social desirability, so further research is needed in order to control for the bias caused by this variable.

## 5. Conclusions

Our findings demonstrated the need to include gender-based violence as a topic in university training and prevention schemes, in order to ensure adequate awareness among students. Universities have a social responsibility to train professionals with an accurate knowledge of the issue. Although our results suggested that significant progress has been made, particularly in terms of an increased rejection of victim blaming beliefs, other myths associated with violence normalization and perpetrator’s exoneration are still deeply-rooted in society. Those myths that question and undervalue institutional and professional victim-support policies, resources, and services are particularly concerning due to their high level of acceptance among the college men and even also the high percentage of students who take refuge in the intermediate position. Our findings revealed the importance of including IPVAW awareness training in the university curriculum, particularly in those disciplines responsible for training the future professionals who will one day work in this area. It is essential to maintain awareness-raising measures aimed at future healthcare professionals, but it is also particularly necessary to include specialized training for future professionals in the fields of law, social sciences, and engineering to ensure that these false beliefs about intimate partner violence against women do not negatively impact their future professional activities.

## Figures and Tables

**Table 1:** Means, standard deviations, and intercorrelations between all study variables.

Scale	Women		Men		1	2	3	4	5	6
	M	SD	M	SD						
1. MV	1.85	0.67	2.47	0.86	-	0.28 **	0.25 **	0.30 **	0.60 **	0.32 **
2. BV	1.23	0.39	1.41	0.49	0.35 **	-	0.20 **	0.16 **	0.33 **	0.32 **
3. EP	2.19	0.73	2.35	0.71	0.26 **	0.18 **	-	18 **	0.30 **	0.34 **
4. NV	2.52	0.86	2.76	0.90	0.32 **	0.25 **	0.15 **	-	0.27 **	0.15 **
5. HS	1.71	0.64	2.46	0.87	0.75 **	0.35 **	0.32 **	0.33 **	-	0.57 **
6. BS	1.80	0.60	2.17	0.71	0.34 **	0.31 **	0.36 **	0.01 *	0.54 **	-

Note. * *p* < 0.05. ** *p* < 0.001. The sample size was 848 women and 672 men (11 missing). Correlations for women are presented above the diagonal, and for men, below. MV: minimizing violence; BV: blaming the victim; EP: exonerating the perpetrator; NV: normalizing violence; HS: hostile sexism; BS: benevolent sexism.

**Table 2:** Differences in the acceptance of myths about IPVAW by gender.

	Total Sample N = 1520 *M* (*SD*)	Women N = 848 *M* (*SD*)	Men N = 672 *M* (*SD*)	*t* (*p*)	Effect Size (Cohen’s *d*)
Item 7	2.01 (1.16)	1.62 (0.92)	2.51 (1.24)	15.52 (<0.001)	0.82
Item 9	2.18 (1.03)	2.01 (1.00)	2.38 (1.01)	6.98 (<0.001)	0.37
Item 13	2.14 (1.04)	1.92 (0.96)	2.42 (1.07)	9.67 (<0.001)	0.50
Item 14	2.18 (1.16)	1.88 (1.05)	2.57 (1.18)	11.84 (<0.001)	0.62
MV	2.12 (0.82)	1.85 (0.67)	2.47 (0.86)	15.17 (<0.001)	0.37
Item 2	1.12 (0.41)	1.07 (0.30)	1.20 (0.51)	5.79 (<0.001)	0.31
Item 4	1.30 (0.60)	1.23 (0.56)	1.38 (0.65)	4.66 (<0.001)	0.25
Item 5	1.38 (0.80)	1.24 (0.70)	1.55 (0.89)	7.20 (<0.001)	0.39
Item 8	1.45 (0.89)	1.40 (0.87)	1.51 (0.92)	2.38 (0.017)	0.12
BV	1.31 (0.44)	1.23 (0.39)	1.41 (0.49)	7.41 (<0.001)	0.19
Item 1	1.60 (1.00)	1.53 (0.97)	1.70 (1.04)	3.36 (<0.001)	0.17
Item 3	3.44 (1.49)	3.36 (0.15)	3.53 (1.42)	2.19 (0.028)	0.11
Item 6	2.22 (1.00)	2.20 (1.00)	2.23 (0.99)	0.56 (0.573)	–
Item 15	1.81 (0.90)	1.69 (0.86)	1.96 (0.92)	5.96 (<0.001)	0.30
EP	2.26 (0.73)	2.19 (0.73)	2.35 (0.71)	4.07 (<0.001)	0.11
Item 10	3.29 (1.20)	3.24 (1.21)	3.34 (1.19)	1.74 (0.083)	–
Item 11	2.69 (1.22)	2.54 (1.22)	2.90 (1.20)	5.76 (<0.001)	0.30
Item 12	1.91 (1.03)	1.80 (0.98)	2.04 (1.09)	4.57 (<0.001)	0.23
NV	2.63 (0.88)	2.52 (0.86)	2.76 (0.90)	5.33 (<0.001)	0.14
MIPVAW	2.04 (0.48)	1.91 (0.43)	2.21 (0.49)	12.00 (<0.001)	0.65

Note. MV: minimizing violence; BV: blaming the victim; EP: exonerating the perpetrator; NV: normalizing violence; MIPVAW: Myths about IPVAW. This sample does not include 11 participants who did not identify themselves as men or women.

## Data Availability

The data that support the findings of this study are available from the corresponding author, Angeles Rebollo-Catalan, upon reasonable request.
